# Intra-oral scan segmentation using deep learning

**DOI:** 10.1186/s12903-023-03362-8

**Published:** 2023-09-05

**Authors:** Shankeeth Vinayahalingam, Steven Kempers, Julian Schoep, Tzu-Ming Harry Hsu, David Anssari Moin, Bram van Ginneken, Tabea Flügge, Marcel Hanisch, Tong Xi

**Affiliations:** 1 Department of Oral and Maxillofacial Surgery, Radboud University Nijmegen Medical Centre, Nijmegen, the Netherlands; 2 Department of Artificial Intelligence, Radboud University, Nijmegen, the Netherlands; 3 Department of Oral and Maxillofacial Surgery, Universitätsklinikum Münster, Münster, Germany; 4 Promaton Co. Ltd, 1076 GR Amsterdam, The Netherlands; 5 MIT Computer Science & Artificial Intelligence Laboratory, 32 Vassar St, Cambridge, MA 02139 USA; 6 Department of Radiology, Radboud University Nijmegen Medical Centre, Nijmegen, the Netherlands; 7 Charité – Universitätsmedizin Berlin, corporate member of Freie Universität Berlin and Humboldt-Universität Zu Berlin, Department of Oral and Maxillofacial Surgery, Hindenburgdamm 30, 12203 Berlin, Germany

**Keywords:** Deep learning, Artificial intelligence, Intra-oral scan, Computer-assisted planning, Digital imaging

## Abstract

**Objective:**

Intra-oral scans and gypsum cast scans (OS) are widely used in orthodontics, prosthetics, implantology, and orthognathic surgery to plan patient-specific treatments, which require teeth segmentations with high accuracy and resolution. Manual teeth segmentation, the gold standard up until now, is time-consuming, tedious, and observer-dependent. This study aims to develop an automated teeth segmentation and labeling system using deep learning.

**Material and methods:**

As a reference, 1750 OS were manually segmented and labeled. A deep-learning approach based on PointCNN and 3D U-net in combination with a rule-based heuristic algorithm and a combinatorial search algorithm was trained and validated on 1400 OS. Subsequently, the trained algorithm was applied to a test set consisting of 350 OS. The intersection over union (IoU), as a measure of accuracy, was calculated to quantify the degree of similarity between the annotated ground truth and the model predictions.

**Results:**

The model achieved accurate teeth segmentations with a mean IoU score of 0.915. The FDI labels of the teeth were predicted with a mean accuracy of 0.894. The optical inspection showed excellent position agreements between the automatically and manually segmented teeth components. Minor flaws were mostly seen at the edges.

**Conclusion:**

The proposed method forms a promising foundation for time-effective and observer-independent teeth segmentation and labeling on intra-oral scans.

**Clinical significance:**

Deep learning may assist clinicians in virtual treatment planning in orthodontics, prosthetics, implantology, and orthognathic surgery. The impact of using such models in clinical practice should be explored.

## Introduction

In recent years, the development of digital dentistry has revolutionized the dental field [1]. 3D virtual treatment planning and subsequent computer-aided design/computer-aided manufacturing of occlusal splints, surgical guides, and prothesis are increasingly being implemented in the clinical workflow [2–4]. One commonly used imaging technique within the scope of virtual treatment planning is the intra-oral scan, which provides a 3D mesh of the dentition [1].

These 3D meshes (OS) are widely used in orthodontics, prosthetics, implantology, and orthognathic surgery to plan patient-specific treatments, which require teeth segmentations with high accuracy and resolution [3]. Teeth segmentations aim to separate and classify the 3D mesh of the dental arch into different teeth following the FDI standard so that each individual tooth position can be rearranged and realigned accordingly. Manual teeth segmentation, the gold standard up until now, is time-consuming, tedious, and observer-dependent [5]. To be able to implement digital models as a clinical standard, fully-automated segmentation of teeth with high accuracy is required [6]. This remains challenging due to the positional variations, shape alterations, size abnormalities, and differences in the number of teeth between individuals [6].

Recently, artificial intelligence (AI) and more specifically deep learning (e.g. convolutional neural network (CNN)) has shown superior segmentation performance compared to geometry-based approaches, mainly due to task-oriented extraction and fusion of local details and semantic information [7].

In dentistry, CNNs have been successfully applied to detect carious lesions [8], periodontal lesions [9], cysts [10], and tumors [11] and even surpassed the detection performance of experienced clinicians in certain conditions [12]. Further deep learning based applications are the difficulty assessment of endodontic treatment [13], prediction of extraction difficulty for mandibular third molars [14], skeletal classification [15], soft tissue prediction [16], and root morphology evaluation [17].

The capability of CNNs to automatically segment teeth on OS(s) were explored in different studies [6, 18–23]. However, these CNNs are black boxes and lack interpretability [24]. Clinicians and patients demonstrate reticence in confiding and adopting AI systems, which are not transparent, understandable, and explainable [25, 26]. For this reason, this study aimed to develop an explainable detection, segmentation, and FDI labeling system using deep learning as a fundamental basis for improved and more automated treatment planning in dentistry.

## Material and methods

### Data

In the present study 1750 3D scans (875 maxilla, 875 mandible) from 875 patients were randomly collected from different clinics in the Netherlands. The accumulated 3D scans (intra-oral scan and gypsum casts scan) were acquired with 3Shape Trios Move, 3Shape D500 (3shape, Copenhagen, Denmark), DW 3Series + , DW 7Series, DW 3Series, and DW 5Series (Dental Wings, Montreal, Canada). This study was conducted in accordance with the code of ethics of the World Medical Association (Declaration of Helsinki) and the ICH-GCP. The Institutional Review Board, Commissie Mensgebonden Onderzoek Radboudumc, Nijmegen, The Netherlands approved the study and granted the approval that informed consent was not required as all image data were anonymized and de-identified before analysis (decision no. 2021–13253).

### Data annotation

The OS were mesh-wise annotated (teeth and gingiva) by different clinicians independently and in duplicate using the brush mode in Meshmixer (Autodesk, San Rafael, United States). Each triangle surface could only belong to one of the two classes. All segmented and labeled OS were subsequently reviewed and revised by two different clinicians (MH, DM). Each of the clinicians and reviewers was instructed and calibrated in the segmentation task using a standardized protocol before the annotation and reviewing process. The definitive dataset was constructed from all annotated meshes.

The training boxes were calculated based on the mesh-wise annotation. For each tooth in the OS, the training box is determined by computing the minimum 3D bounding box around the tooth’s points.

### The model

**Fig. 1:**
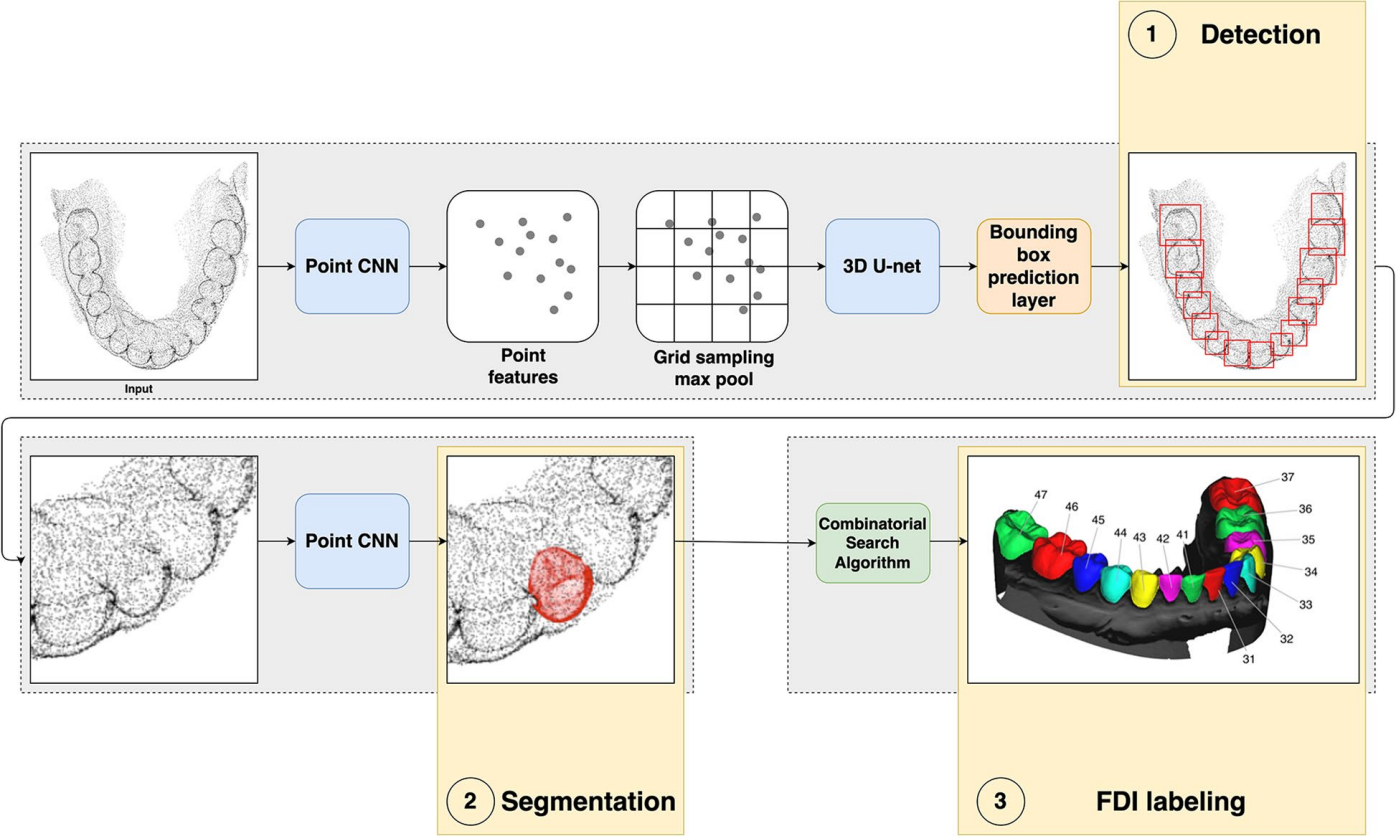
The workflow of detection, segmentation and labeling process

The OS detection, segmentation, and labeling process included three parts: the detection module, the segmentation module, and the labeling algorithm (Fig. 1).

#### The detection module

The detection module was comprised of two different CNNs: 1). PointCNN [27] and 2). 3D-Unet [28].

PointCNN is an architecture tailored for point cloud processing tasks, operating on unordered point sets. This architecture incorporates a learnable permutation invariant operation that efficiently gathers and aggregates local features from neighboring points, facilitating effective feature learning while preserving the inherent structure of the point cloud. The 3D-Unet is a modified version of the U-net architecture. It consists of an encoder, which down-samples the input volume to capture hierarchical features, skip connections to preserve spatial information, and a decoder, which up-samples the feature.

An OS was uniformly downsampled to 30,000 vertices. The PointCNN acted as an initial feature extractor on the downsampled OS. The PointCNN encodes an OS to a point cloud where each vertex is represented with 256 features. This downsampled point cloud is transformed to a Cartesian grid by max pooling the features of all points in one grid cell. The distributed surface points on the entire grid domain were fed forward to the 3D-Unet. In this stage, the model estimated the bounding box dimensions and its central position. The final aggregated bounding box proposals were used as inputs for the segmentation task [21].

#### The segmentation module

The points pertaining to a detected tooth were extracted from the OS by expanding the tooth’s bounding box and uniformly sampling 8192 points within the expanded volume. A PointCNN was used in the segmentation module. Each point located inside the 3D bounding box was binary classified as a tooth or gingiva.

#### The labeling algorithm

The $N$ detected teeth from the model were assigned to $C=32$ FDI numbers. This was carried out by filling in an assignment matrix $E\in {\left\{0, 1\right\}}^{N\times C}$ from a mathematical perspective. The solution space was immense; hence, efficient heuristics were required to reduce the space effectively. For this reason, a penalty function $f\left(E\right)$ and an associated exploration strategy space $\Omega$ were formulated. The resulting assignment $\underline{E}=arg\underset{E\in \Omega }{min} f\left(E\right)$ would be the one assignment that minimized the penalty.

The post-processing was carried out in multiple stages, each refining upon the previous assignment, exploring the assignments that were similar to the existing one. Prior to post-processing, the center of mass (COM) of each detection $n$, $CO{M}_{n}$, was calculated by extracting the center of the associated segmentation mask. The mean of all COMs was represented by $CO{M}_{\odot }$, the axial component of which, ${COM}_{\odot }^{z}$, roughly acted as a watershed between two half jaws. The COMs are used extensively in subsequent penalty calculations.

As a first stage, $E$ was greedily assigned to minimize ${\underline{E}}_{1}=arg\underset{E\in {\Omega }_{Greedy}}{min} {f}_{1}\left(E\right)$,

$${f}_{1}\left(E\right)={f}_{11}\left(E\right)+{{\lambda }_{12}f}_{12}\left(E\right)+{\lambda }_{13}{f}_{13}\left(E\right)$$

$$={\sum }_{c}\underset{}{max}\left({\sum }_{n}{e}_{nc}-1, 0\right)$$

$$+{\lambda }_{12}{\sum }_{n}\left({\sum }_{c\in\;Upper\;Jaw}{e}_{nc}\cdot 1\left[{COM}_{n}^{z}<{COM}_{\odot }^{z}\right]+{\sum }_{c\in\;Lower\;Jaw}{e}_{nc}\cdot 1\left[{COM}_{\odot }^{z}<{COM}_{n}^{z}\right]\right)+{\lambda }_{13}{\sum }_{n}\underset{}{max}\left(1-{\sum }_{c}{e}_{nc}, 0\right),$$

where ${f}_{11}$ wished to have all FDI numbers assigned to an unique object, ${f}_{12}$ aimed to have detections assigned to the right jaw, and ${f}_{13}$ reduced the count of unassigned detections to a minimum. $\lambda$’s were weights, and were set at ${\lambda }_{12}=0.1$ and ${\lambda }_{13}=0.01$. For the second stage, a permutated space of ${\underline{E}}_{1}$ was explored where the assigned detections remained assigned in each jaw while having a possible permutation of FDI numbers (i.e., ${\sum }_{c}{e}_{nc}$ stays constant $\forall n$). This step encourages the FDI numbers to become sorted.

${\underline{E}}_{2}=arg\underset{E\in {\Omega }_{Permutation}\left({\underline{E}}_{1}\right)}{min} {f}_{2}\left(E\right)$ is minimized, where

$$f_2\left(E\right)=\sum\limits_{n_1,n_2}\sum\limits_{c_1,c_2\in UpperJaw}e_{n_1c_1}\cdot e_{n_2c_2}\cdot1\left[\left({COM}_{n_1}^x>{COM}_{n_2}^x\right)\oplus\left(c_1>c_2\right)\right]$$

$$+\sum\limits_{n_1,n_2}\sum\limits_{c_1,c_2\in LowerJaw}e_{n_1c_1}\cdot e_{n_2c_2}\cdot1\left[\left({COM}_{n_1}^x>{COM}_{n_2}^x\right)\oplus\left(c_1>c_2\right)\right]$$

In the formula, $CO{M}^{x}$ ($x$ went from left to right for the patient) was enforced to grow monotonically while the FDI number increased. $\oplus$ denotes exclusive or.

Finally, the sorted relationship in ${\underline{E}}_{2}$ was retained, but allowed insertion/removal of blank assignments and minimize ${\underline{E}}_{3}=arg\underset{E\in {\Omega }_{Sorted}\left({\underline{E}}_{2}\right)}{min} {f}_{3}\left(E\right)$, where

$${f}_{3}\left(E\right)={\sum }_{{n}_{1},{n}_{2}}{\sum }_{{c}_{1},{c}_{2}\in Upper Jaw}{e}_{{n}_{1}{c}_{1}}\cdot {e}_{{n}_{2}{c}_{2}}\cdot {\left(\left|CO{M}_{{n}_{1}}-CO{M}_{{n}_{2}}\right|-{D}_{{c}_{1}{c}_{2}}\right)}.$$

The purpose of the penalty was to minimize the difference between the distance of a pair of teeth and their corresponding predetermined distance parameter. The distance, ${D}_{{c}_{1}{c}_{2}}$ was a prior matrix based on the training dataset that represented the mean of distances (in millimeters) across the whole set.

The resulting assignment after three stages of refinement, ${\underline{E}}_{3}$, would then be used for subsequent analysis.

### Model training

The annotated 3D meshes were randomly split into three sets of OS(s), 1224 for training (612 patients), 176 for validation (88 patients), and 350 for testing (175 patients). The validation set was used to evaluate the model convergence during training, while the hold-out test set was used to evaluate the model performance after training. Data augmentation techniques such as shuffle points, feature normalization, flips, and rotations around the z-axis were employed on the training set.

The detection module was trained over 180 epochs with a learning rate decay of 0.8 while the segmentation module was trained for 50 epochs with a learning rate decay of 1. The applied batch size was one for the detection module with 30,000 vertices and batch size three for the segmentation module with 8192 vertices. Weight decay of 0.0001 and early stopping were applied for both modules. Both modules used the Adam optimizer at a learning rate of 0.001. No momentum or gradient clipping were applied. The binary cross-entropy loss function was applied for the segmentation module. The detection module used a multi-task loss function consisting of binary cross-entropy loss and IoU loss. The model was implemented in TensorFlow 1.8 and trained on an NVIDIA ® V100 Tensor Core GPU 16G.

### Statistical analysis

The model predictions on the test set were compared to the expert annotations. Object detection, instance segmentation and FDI labeling metrics were reported as follows for the test set: accuracy = $\frac{TP+TN}{TP+TN+FP+FN}$, precision = $\frac{TP}{TP+FP}$, recall = $\frac{TP}{TP+FN}$ and intersection over union (IoU) = $\frac{TP}{TP+FP+FN}$. TP, TN, FP and FN denote true positives, true negatives, false positives and false negatives, respectively [5].

## Results

The model achieved high detection accuracies on the test set (350 OS(s)) with a precision of 0.994, recall of 0.988, and average bounding box IoU of 0.806 (Table 1). The bounding box IoU for individual teeth ranged from 0.718 to 0.873. The detection model had, in total, 54 missed detections and 29 false-positive detections.

**Table 1:** Precision, recall, and Intersection over Union (IoU) of the detections

**Tooth**	**Precision**	**Recall**	**IoU**_**BoundingBox**_
**11**	1.000	1.000	0.848
**12**	1.000	0.994	0.806
**13**	0.988	0.942	0.831
**14**	0.983	1.000	0.847
**15**	0.942	1.000	0.819
**16**	0.994	1.000	0.863
**17**	0.969	0.976	0.810
**18**	0.263	0.833	0.801
**21**	1.000	1.000	0.836
**22**	1.000	1.000	0.778
**23**	0.959	0.959	0.810
**24**	0.989	1.000	0.843
**25**	0.926	0.994	0.821
**26**	0.983	0.994	0.873
**27**	0.969	0.992	0.821
**28**	1.000	1.000	0.718
**31**	1.000	1.000	0.742
**32**	1.000	1.000	0.796
**33**	0.988	0.988	0.796
**34**	0.965	1.000	0.814
**35**	0.868	0.986	0.800
**36**	0.994	1.000	0.818
**37**	0.943	0.935	0.765
**38**	0.429	0.500	0.824
**41**	0.994	0.988	0.727
**42**	0.994	1.000	0.764
**43**	0.976	0.982	0.754
**44**	0.988	0.994	0.801
**45**	0.900	0.994	0.812
**46**	0.994	0.994	0.814
**47**	0.943	0.951	0.767
**48**	0.750	1.000	0.743

Considering a successful detection, the model achieved teeth segmentations with an average IoU score of 0.915 (Table 2). The segmentation IoU, recall, precision and accuracy for individual teeth ranged from 0.792 to 0.948, 0.847 to 0.993, 0.880 to 0.966, and 0.989 to 0.998, respectively.

**Table 2:** Accuracy, precision, recall, and Intersection over Union (IoU) of the OS segmentations

**Tooth**	**Accuracy**	**Precision**	**Recall**	**IoU**_**Mask**_
**11**	0.997	0.935	0.990	0.926
**12**	0.998	0.923	0.992	0.916
**13**	0.998	0.931	0.991	0.923
**14**	0.997	0.935	0.993	0.929
**15**	0.998	0.941	0.992	0.933
**16**	0.997	0.961	0.987	0.948
**17**	0.996	0.946	0.959	0.909
**18**	0.998	0.966	0.971	0.939
**21**	0.997	0.931	0.988	0.921
**22**	0,997	0.916	0.993	0.910
**23**	0.997	0.911	0.993	0.905
**24**	0.997	0.937	0.992	0.929
**25**	0.997	0.937	0.992	0.929
**26**	0.997	0.955	0.989	0.945
**27**	0.995	0.940	0.935	0.881
**28**	0.997	0.880	0.983	0.867
**31**	0.996	0.899	0.989	0.890
**32**	0.997	0.919	0.990	0.909
**33**	0.997	0.927	0.991	0.919
**34**	0.997	0.932	0.993	0.926
**35**	0,997	0.937	0.992	0.931
**36**	0.994	0.959	0,965	0.926
**37**	0.990	0.941	0.887	0.839
**38**	0.998	0.955	0.992	0.948
**41**	0.997	0.906	0.989	0.896
**42**	0.997	0.918	0.989	0.908
**43**	0.996	0.915	0.991	0.907
**44**	0.997	0.933	0.992	0.926
**45**	0.997	0.940	0.991	0.932
**46**	0.994	0.958	0.974	0.933
**47**	0.989	0.935	0.876	0.824
**48**	0.990	0.934	0.847	0.792

**Fig. 2:**
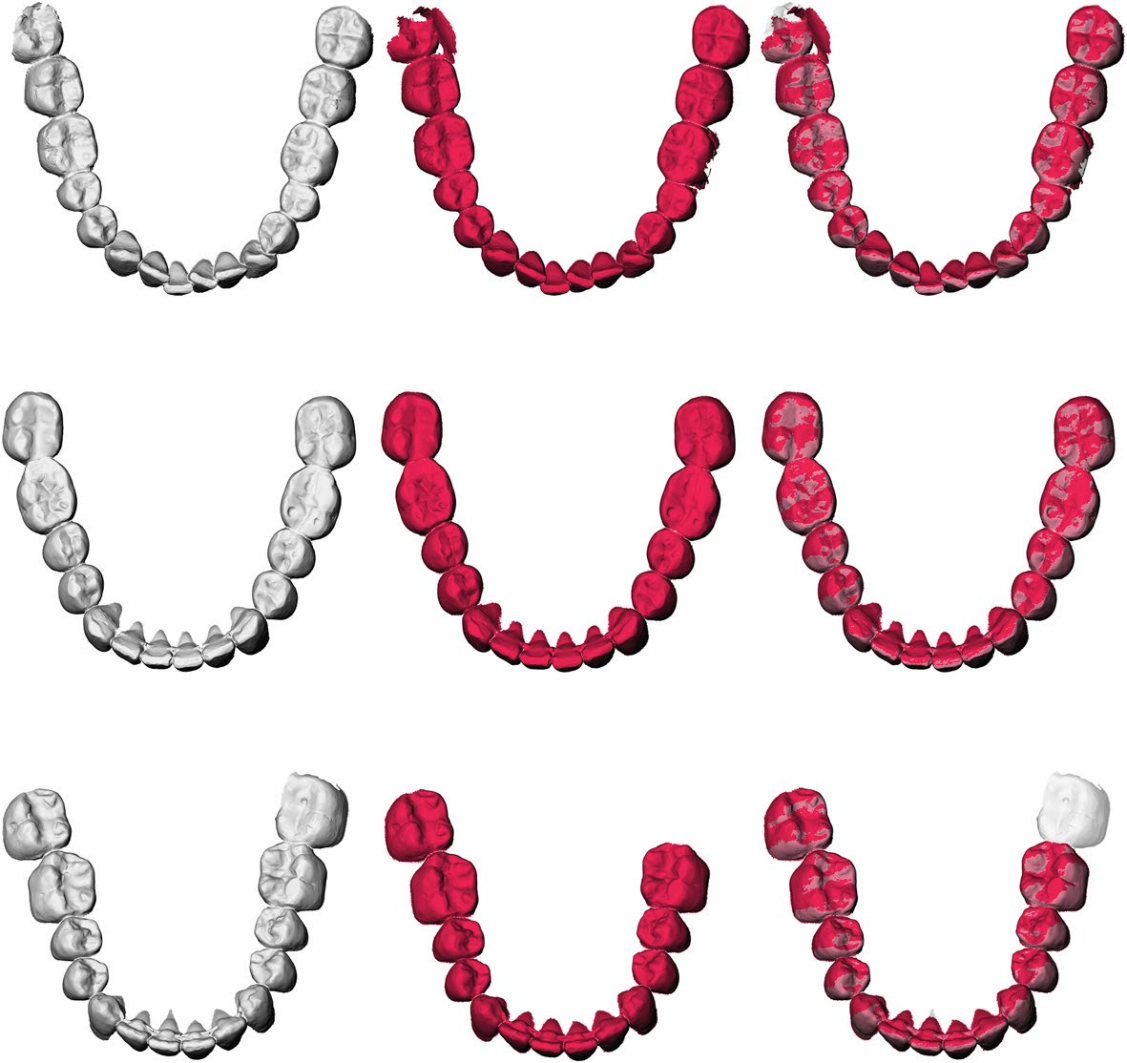
Overview of mandible teeth segmentations; left: manual segmentation; middle: automatic segmentation; right: overlay; one of the two detection errors is illustrated

**Fig. 3:**
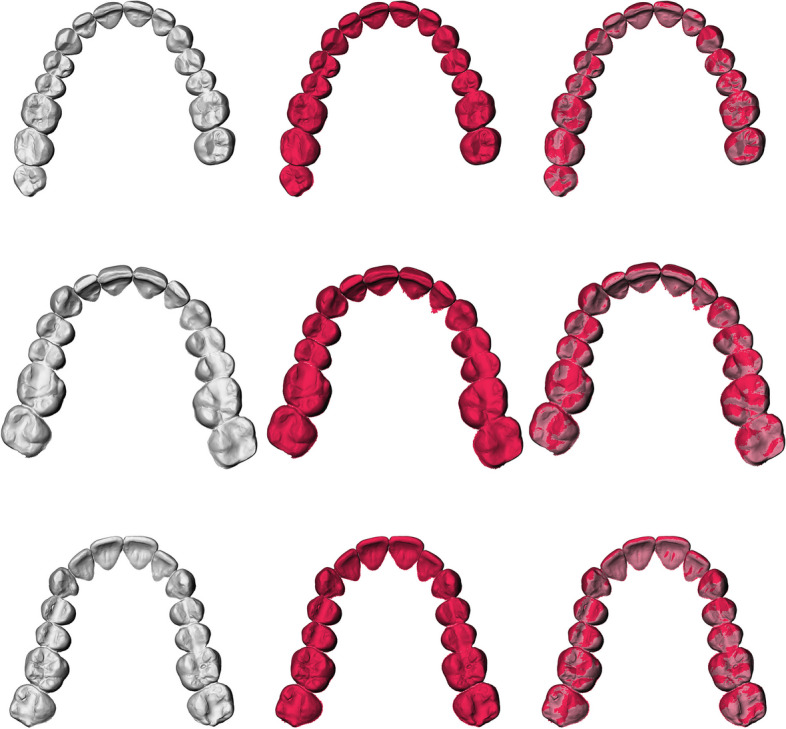
Overview of maxillary teeth segmentations; left: manual segmentation; middle: automatic segmentation; right: overlay

The optical inspection (Figs. 2 and 3) showed excellent position agreements between the automatically and manually segmented teeth components. Minor flaws were mainly seen cervically, and the lowest segmentation and detection accuracies were seen for the third molars.

**Fig. 4:**
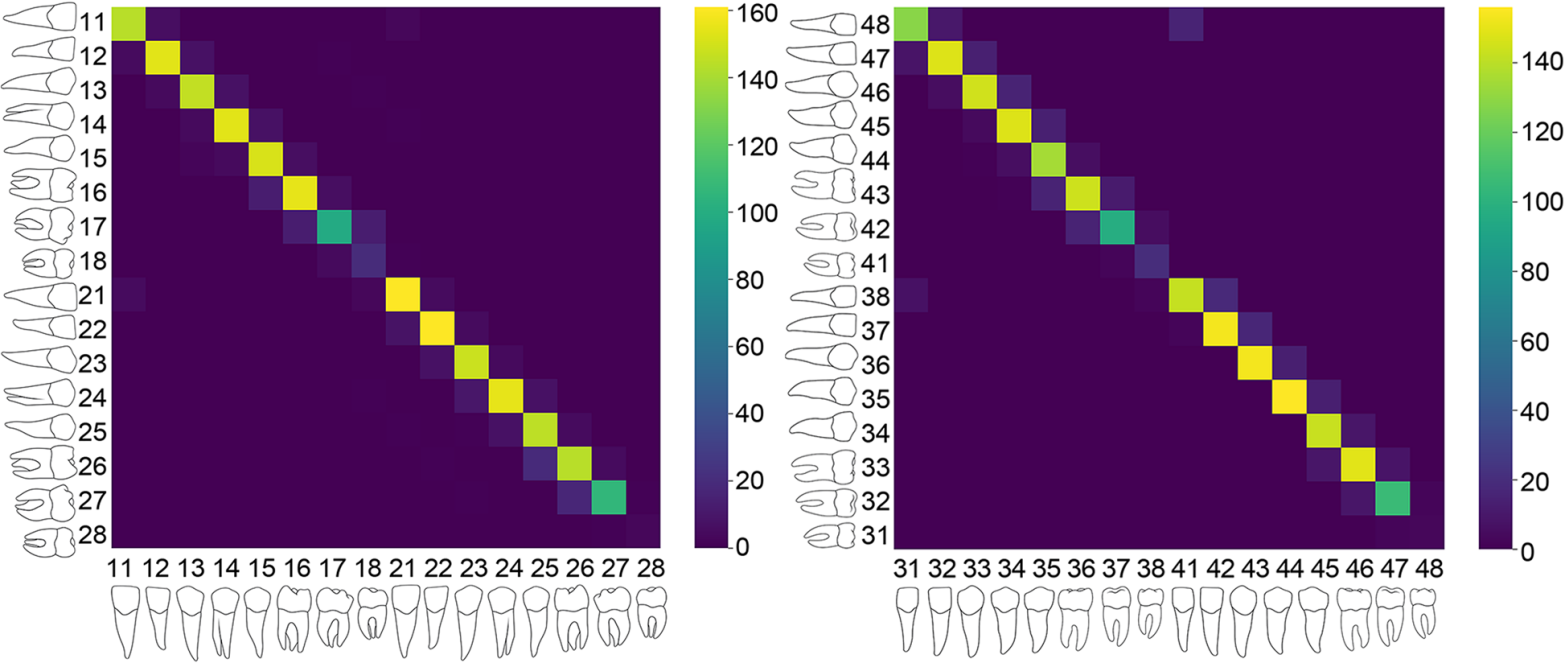
Confusion Matrices show the agreement between actual and predicted classes to indicate labeling accuracy, and brighter cells signify a higher class agreement. The left and right matrices display the model performance in the maxilla and mandible, respectively

The FDI labels of the teeth were predicted with an accuracy of 0.894 (Table 3). The accuracy range for individual teeth was between 0.6 and 1. Figure 4 illustrates the confusion matrices for the upper and lower jaw.

**Table 3:** Accuracy of the FDI numeration

**Tooth**	**Accuracy**
**11**	0.944
**12**	0.943
**13**	0.944
**14**	0.947
**15**	0.945
**16**	0.902
**17**	0.797
**18**	0,800
**21**	0.938
**22**	0.938
**23**	0.944
**24**	0.913
**25**	0.926
**26**	0.871
**27**	0.873
**28**	0.600
**31**	0.850
**32**	0.879
**33**	0.892
**34**	0.898
**35**	0.931
**36**	0.849
**37**	0.843
**38**	1.000
**41**	0.847
**42**	0.884
**43**	0.916
**44**	0.918
**45**	0.941
**46**	0.905
**47**	0.914
**48**	0.667

## Discussion

The field of AI in dentistry is rapidly advancing and holds great potential for significant contributions to dental practices in the near future [26–29]. Chen et al. categorized AI systems into three types: pre-appointment, inter-appointment, and post-appointment systems (30). These systems can aid in patient management by analyzing their needs and risks before appointments, assisting in diagnosis, treatment planning, and outcome prediction during appointments, and supporting labor work such as prosthodontics design and treatment evaluation after appointments [18]. Particularly, 3D treatment planning can be time-consuming and laborious, but with the help of automated assistance, it can become more time-efficient, leading to a more cost-effective 3D treatment planning process [6]. In this study, the researchers evaluated the performance of a deep learning model for automating 3D teeth detection, segmentation, and FDI labeling on 3D meshes.

In dentistry, different studies have applied deep learning models for segmentation on 3D meshes [6, 20–23]. Lian et al. introduced a mesh-based graph neural network for teeth segmentation with an F1-score of 0.981 [23]. Zhao et al. used a graph attentional convolution network with a local spatial augmentation module for segmentation and achieved a mean IoU of 0.871 [22]. Zanjani et al. proposed a volumetric anchor-based region proposal network for teeth point cloud detection and segmentation with a mean IoU of 0.98 [21]. Cui et al. applied a two-stage network architecture for tooth centroid extraction using a distance-aware voting scheme and segmentation with an F1-score of 0.942 [20]. Similarly, Hao et al. proposed a two-module approach. The segmentation module generated a fine-grained segmentation, whereas the canary module autocorrected the segmentation based on confidence evaluation. Hao et al. reported a mean IoU of 0.936 and 0.942 for mandible and maxillary teeth, respectively [6].

The number of studies reporting the classification and semantic labeling accuracies of each tooth is yet limited [18, 19]. Tian et al. employed a 3D CNN using a sparse voxel octree for teeth classification with an accuracy of 0.881 [18]. Ma et al. proposed a deep learning network to predict the semantic label of each 3D tooth model based on spatial relationship features. The proposed SRF-Net achieved a classification accuracy of 0.9386 [19].

It is important to recognize that the performance of deep learning models relies heavily on factors such as the dataset, hyperparameters, and architecture involved [8]. One key obstacle to reproducing and validating previous results is the restricted accessibility of the datasets used, stemming from privacy concerns. Furthermore, the considerable variation in training and test sets sizes across different studies makes it difficult to draw direct comparisons. The lack of clarity regarding data representativeness further compounds the issue.

Moreover, attempting to reproduce complex computational pipelines based solely on textual descriptions without access to the source code becomes a subjective and challenging task (31). The inadequate description of training pipelines, essential hyperparameters, and current software dependencies undermines the transparency and reproducibility of earlier findings. Given these limitations, it's essential to approach any direct comparison of previous segmentation and labeling results with caution [5].

Even though previous studies achieved remarkable results, the models are regarded as black boxes lacking explicit declarative knowledge representation. Generating the underlying explanatory structures is essential in the medical domain to provide clinicians with a transparent, understandable, and explainable system [29]. The current study made the results re-traceable on demand using a hierarchical three-step plug-and-play pipeline. This pipeline allows clinicians to verify the immediate results of each module before proceeding further. In case the detection module fails to detect a tooth, the clinician can correct the mistake immediately and proceed to the subsequent module. This stop-and-go approach ensures an efficient workflow while maintaining high precision and explainability. Another advantage of this plug-and-play pipeline is the interchangeability of the different modules. The detection and segmentation modules can be exchanged with alternative model architectures without much difficulties.

The segmentation IoU scores ranged between 0.792 and 0.948. Furthermore, each tooth was classified and labeled with an accuracy between 0.6 and 1. The lowest segmentation and labeling accuracies were seen for third molars. Hierarchical concatenation of different deep learning models and post-processing heuristics have the disadvantage that the errors in the different modules are cumulative. In other words, inaccuracies in the detection module will affect the segmentation module and the FDI labeling algorithm. However, this shortcoming can be neglected if the pipeline is interactively used with the clinicians.

Although our proposed model has achieved clinically applicable results, it has some limitations. Wisdom teeth, supernumerary teeth, or crowded teeth impede the segmentation and labeling accuracies. Most failure cases are related to rare or complicated dental morphologies [6, 7, 18–20]. Without real-world integration, deep learning models are bound to the limits of the training set and validation set. Furthermore, extensive model comparisons are required to choose the optimal model architectures for the respective modules (e.g., Point-RCNN for the detection module). Future studies should focus on further automation of 3D treatment planning steps, such as automated crown design and automated alignment of intra-oral scans and cone-beam computed tomography.

The proposed model is currently clinically used for orthodontic treatment planning. The constant error reductions and adaptions to real-world cases will further enhance the current model. The successful implementation of this approach in daily clinical practice will also further reduce the risks of limited robustness, generalizability, and reproducibility.

## Conclusion

In conclusion, our proposed method achieved accurate teeth segmentations with a mean IoU score of 0.915. The FDI labels of the teeth were predicted with a mean accuracy of 0.894. This forms a promising foundation for time-effective and observer-independent teeth segmentation and labeling on intra-oral scans.

## Data Availability

The datasets used and/or analyzed during the current study are available from the corresponding author upon reasonable request.
